# Dicarbonyl­chlorido(phen­oxy­thio­carbonyl-κ^2^ *C*,*S*)bis­(triphenyl­phosphane-κ*P*)molybdenum(II)

**DOI:** 10.1107/S1600536810052530

**Published:** 2010-12-24

**Authors:** Gene-Hsiang Lee, Hsiao-Fen Wang, Kuang-Hway Yih, Shou-Ling Huang

**Affiliations:** a Instrumentation Center, College of Science, National Taiwan University, Taipei 106, Taiwan; b Department of Applied Cosmetology, Hungkuang University, Shalu 433, Taichung, Taiwan

## Abstract

In the title complex, [Mo(C_7_H_5_OS)Cl(C_18_H_15_P)_2_(CO)_2_], the geometry around the metal atom is a capped octa­hedron. The phen­oxy­thio­carbonyl ligand coordinates the Mo^II^ atom through the C and S atoms. A one-dimensional structure is formed by π–π inter­molecular inter­actions and a supra­molecular aggregation is determined by inter­molecular C—H⋯O, C—H⋯Cl, C—H⋯π(arene) hydrogen bonds and CO⋯π(arene) inter­actions [O⋯centroid distances = 3.485 (4) and 3.722 (3) Å].

## Related literature

For the use of metallocarb­oxy­lic acids as inter­mediates in the homogeneous catalysis of the water gas shift reaction, see: Yoshida *et al.* (1978 ▶). For *O*-Aryl thio­carbonate, benzoxazoline-2-thione, chromene-2-thione and *N*,*N*-dimethyl­thio­carb­amate metal complexes, see: Chen *et al.* (1978 ▶); McFarlane *et al.* (1998 ▶); Zheng *et al.* (2006 ▶) and Zhang & Shi (2004 ▶), respectively. For phen­oxy­lcarbonyl metal complexes, see: Anderson *et al.* (2001 ▶). We are inter­ested in the synthesis of dithio­carbamate, pyridine-2-thio­nate (Yih *et al.*, 2010 ▶) and *N*,*N*-dimethyl­dithio­carbarmoyl (Yih & Lee, 2010 ▶) metal complexes. For a phen­oxy­thio­carbon­yl–palladium complex, see: Yih & Lee (2004 ▶). For C—H⋯O inter­actions, see: Strasser *et al.* (2009 ▶); Arumugam *et al.* (2010 ▶). For C—H⋯π inter­actions, see: Suresh *et al.* (2007 ▶). For π–π inter­actions, see: Bartholomä *et al.* (2009) ▶; Hu *et al.* (2009 ▶). For the C—H⋯Cl inter­actions, see: Shawkataly *et al.* (2010 ▶); Qi *et al.* (2009 ▶). For C—H⋯S inter­actions, see: Asad *et al.* (2010 ▶); Goh *et al.* (2010 ▶). For C–H⋯acceptor inter­actions, see: Steiner (1996 ▶). For typical C—O and C—S bond lengths, see: Huheey (1983 ▶). For Mo—CO and C—O bond lengths in other molybdenum–carbonyl complexes, see: Yih & Lee (2008 ▶) and references therein.
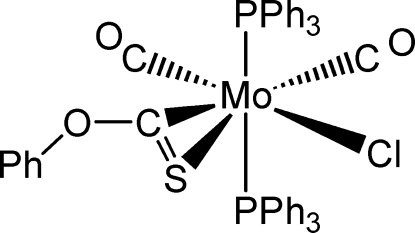


## Experimental

### 

#### Crystal data

[Mo(C_7_H_5_OS)Cl(C_18_H_15_P)_2_(CO)_2_]
*M* *_r_* = 849.12
Triclinic, 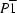

*a* = 10.5685 (10) Å
*b* = 12.5224 (11) Å
*c* = 16.3983 (14) Å
α = 82.088 (2)°
β = 77.476 (2)°
γ = 67.212 (2)°
*V* = 1949.7 (3) Å^3^
*Z* = 2
Mo *K*α radiation
μ = 0.58 mm^−1^
*T* = 150 K
0.16 × 0.15 × 0.10 mm

#### Data collection

Bruker SMART APEX CCD area-detector diffractometer
Absorption correction: multi-scan (*SADABS*; Bruker, 2007 ▶) *T*_min_ = 0.913, *T*_max_ = 0.944
25423 measured reflections
8942 independent reflections
6714 reflections with *I* > 2σ(*I*)
*R*_int_ = 0.075

#### Refinement

*R*[*F* ^2^ > 2σ(*F* ^2^)] = 0.053
*wR*(*F* ^2^) = 0.128
*S* = 1.00
8942 reflections
478 parameters
3 restraints
H-atom parameters constrained
Δρ_max_ = 1.02 e Å^−3^
Δρ_min_ = −0.81 e Å^−3^

### 

Data collection: *SMART* (Bruker, 2007 ▶); cell refinement: *SAINT* (Bruker, 2007 ▶); data reduction: *SAINT*; program(s) used to solve structure: *SHELXS97* (Sheldrick, 2008 ▶); program(s) used to refine structure: *SHELXL97* (Sheldrick, 2008 ▶); molecular graphics: *XP* in *SHELXTL* (Sheldrick, 2008 ▶); software used to prepare material for publication: *SHELXTL*.

## Supplementary Material

Crystal structure: contains datablocks I, global. DOI: 10.1107/S1600536810052530/bg2377sup1.cif

Structure factors: contains datablocks I. DOI: 10.1107/S1600536810052530/bg2377Isup2.hkl

Additional supplementary materials:  crystallographic information; 3D view; checkCIF report

## Figures and Tables

**Table 1:** Hydrogen-bond geometry (Å, °) *Cg*1, *Cg*2, *Cg*3 and *Cg*7 are the centroids of the C4–C9, C10–C15, C16–C21 and C40–C45 rings, respectively.

*D*—H⋯*A*	*D*—H	H⋯*A*	*D*⋯*A*	*D*—H⋯*A*
C23—H23⋯O3	0.95	2.31	3.208 (5)	157
C24—H24⋯O1^i^	0.95	2.58	3.199 (5)	123
C39—H39⋯Cl1	0.95	2.80	3.573 (4)	139
C9—H9⋯*Cg*3	0.95	2.97	3.896 (5)	165
C14—H14⋯*Cg*7^ii^	0.95	2.83	3.663 (5)	147
C20—H20⋯*Cg*1^iii^	0.95	2.97	3.802 (4)	147
C27—H27⋯*Cg*2	0.95	2.84	3.636 (5)	141

Symmetry codes: (i) 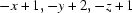; (ii) 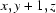; (iii) 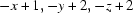.

**Fig. 1.**
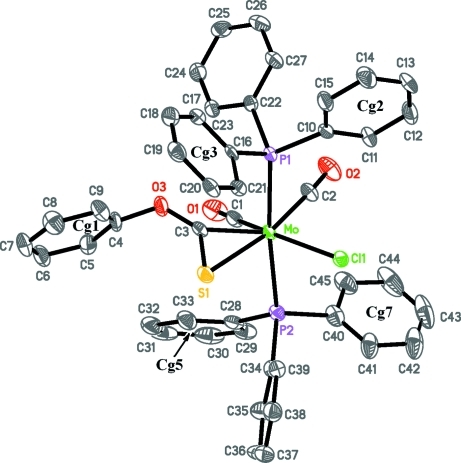
The molecular structure of (I), with atom labels and the 50% probability displacement ellipsoids.

**Fig. 2.**
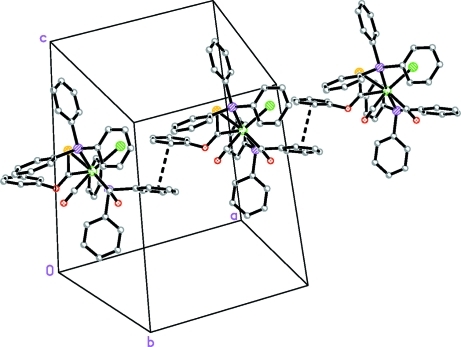
The packing diagram of (I), showing the π-π interaction and 1-D chain structure.

**Fig. 3.**
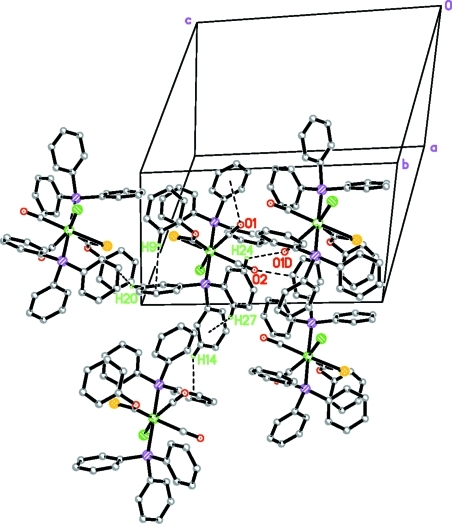
The packing diagram of (I), showing the intermolecular C—H···O, C—H···π(arene) hydrogen bonds and CO···π(arene) interactions.

## supplementary crystallographic information

### Comment

The interest in the M—C(S)OPh moiety is due to its analogy with metallocarboxylic acid esters (M—C(O)OR) and metallocarboxylic acids themselves. Metallocarboxylic acids have been proposed to be the key intermediates in the homogeneous catalysis of the water gas shift reaction (Yoshida *et al.*, 1978). *O*-Aryl thiocarbonate (Chen *et al.*, 1978), benzoxazoline-2-thione (McFarlane *et al.*, 1998), chromene-2-thione (Zheng *et al.*, 2006), and *N*,*N*-dimethylthiocarbamate (Zhang *et al.*, 2004) metal complexes have been reported but few phenoxylcarbonyl metal complexes have been studied (Anderson *et al.*, 2001). We are interested in the synthesis of dithiocarbamate, pyridine-2-thionate (Yih *et al.*, 2010) and *N*,*N*-dimethyldithiocarbarmoyl (Yih & Lee, 2010) metal complexes. To our knowledge, no chelating phenoxythiocarbonyl crystal structure has been described so far.

The molecular structure of the title compound [Mo(CO)_2_(SCOPh)(PPh_3_)_2_Cl], (I),is shown in Fig. 1. The geometry around the metal atom is midway a capped trigonal prism and a capped octahedron. The capped trigonal prism consist of a phosphorus atom, P2, in the unique capping position [Mo1—P2 = 2.5509 (10) Å]. Two carbonyl groups, C1-O1 and C2-O2, Cl1, and the sulfur atom S1 of the phenoxythiocarbonyl ligand are present in the capped quadrilateral face [Mo—C1 = 1.938 (4) Å; Mo—C2 = 1.998 (4) Å; Mo—Cl1 = 2.5160 (9) Å; Mo—S1 = 2.6553 (10) Å] and the phenoxythiocarbonyl ligand is at the unique edge [Mo—S1 = 2.6553 (10) Å; Mo—C3 = 2.025 (4) Å]. In contrast the capped octahedron is made up of C3 in the capping position, C1, S1, and P2 in the capped face, and P1, C2, and Cl1 in the uncapped face. Two PPh_3_ ligands are in *trans* position: P1—Mo—P2, 173.19 (3)°, while the sulfur atom of the phenoxythiocarbonyl ligand, chloride and two carbonyl groups are *trans* to each other: C2—Mo—S1, 170.67 (11)°, C1—Mo—Cl1, 154.93 (12)°. The mean Mo—C—O angle of (I) ( 176.4 (3)° ) shows the group to be essentially linear, similarly to other terminal carbonyls of Mo. The Mo—CO (1.938 (4), 1.998 (4) Å) and C—O (1.163 (4), 1.146 (4) Å) distances are both consistent with the range of values reported for the other molybdenum carbonyl complexes (Yih & Lee, 2008 and references therein). The Mo—C1 bond distance is clearly shorter than that of Mo—C2 due to the larger *trans* influence of the sulfur atom of phenoxythiocarbonyl ligand than that of the chlorine ligand.

Within the SCOPh ligand, the C—S (1.650 (4) Å) and SC—O (1.319 (4) Å) bond distances are typical for C—O and C—S bonds having partial double bond character and are certainly much shorter than typical C—O (1.43 Å) and C—S (1.82 Å) single bonds (Huheey, 1983). The S1—C3—O3 group shows a geometrical environment characteristic of *sp*^2^ hybridization of the carbon atom. In addition, the S1—C3—O3 angle of 129.0 (3)° is larger than that found in the palladium phenoxythiocarbonyl complex (125.2 (6)°) (Yih *et al.*, 2004). To our knowledge, the title complex is the first chelating phenoxythiocarbonyl-metal complex in the literature.

Three weak intramolecular hydrogen bonds and one intermolecular hydrogen bond are present in the structure (Table 1, entries 1-4). In addition, the phenyl ring (C4—C9) of the phenoxythiocarbonyl ligand and a phenyl ring (C10—C15) from the triphenylphosphane are nearly parallel, with an intercentroid distance of 3.938 (3)Å and a shortest inter-ring distance of 3.160 (2) Å. The resulting π-π interaction links molecules into a 1-D chain structure (Fig. 2).Finally, a supramolecular aggregation is determined by four C—H···π(arene) hydrogen bonds (Fig. 3 and Table 1, entries 5-8). The structure also presents some short CO···π(arene) contacts, O1···*Cg*5: 3.485 (4) and O2···*Cg*2^iv^:3.722 (3)Å, ( iv = -*x* + 2,-y_2,-*z* + 1)

In the ^1^H NMR spectrum of (I), 35 protons of the seven phenyl exhibit multiple resonances in the region of δ 7.12–7.73. In the ^13^C{^1^H} NMR spectrum of (I), two triplet resonances appear at δ 229.3 and δ 238.6 with ^2^J_P—C_ = 12.95, 11.95 Hz couplings for the two inequivalent carbonyl groups, respectively. The ^31^P{^1^H} NMR spectrum of (I) shows one resonance at δ 34.2.

It is also noted that  the IR spectrum of the title complex (I) shows four stretching bands, two at 1965, 1891 cm^-1^ for C=O and two at 1483, 1434 cm^-1^ for C-OPh groups. In the FAB mass spectra, the base peak with the typical Mo isotope distribution is in agreement with the [*M*^+^] molecular mass of (I).

### Experimental

The synthesis of the title compound (I) was carried out as follows. PhOCSCl (0.135 g, 1.1 mmol) was added to a flask (100 ml) containing CH_2_Cl_2_ (10 ml) and [Mo(CH_3_CN)_2_(CO)_2_(PPh_3_)_2_] (0.758 g, 1.0 mmol) at room temperature. The color of the solution was changed from yellow to red immediately. The solution was concentrated under vacuum and n-hexane (10 ml) was added to initiate a yellow-brown precipitation. The resulting bright-yellow solid was isolated by filtration (G4), washed with diethyl ether (2 *x* 10 ml) and subsequently dried under vacuum, yielding [Mo(CO)_2_(SCOPh)(PPh_3_)_2_Cl] (0.764 g, 90%). Further purification was accomplished by recrystallization from 1/10 CH_2_Cl_2_/n-hexane. The orange crystals of (I) for X-ray structure analysis were obtained by slow diffusion of n-hexane into the CH_2_Cl_2_ solution of the title compound at room temperature for 3 days. Spectroscopic analysis: ^1^H NMR (CDCl_3_, 298 K, δ, p.p.m.): δ 7.12–7.73 (m, 35H, Ph). ^31^P{^1^H} NMR (CDCl_3_, 298 K, δ, p.p.m.): δ 34.3. ^13^C{^1^H} NMR (CDCl_3_, 298 K, δ, p.p.m.): δ 127.9- 134.2 (m, C of Ph), 159.7 (s, O—Ph), 229.3, 238.6 (t, CO, ^2^J_P—C_ = 12.95, 11.95 Hz). MS (m/*z*): 850 (*M*^+^). Anal. Calcd for C_45_H_35_ClO_3_P_2_SMo: C, 63.65; H, 4.16. Found: C, 63.50; H, 4.05.

### Refinement

H atoms were positioned geometrically and refined using a riding model, with C—H = 0.95Å and with *U*_iso_(H) = 1.2 times *U*_eq_(C).

### Crystal data

**Table:**

[Mo(C_7_H_5_OS)Cl(C_18_H_15_P)_2_(CO)_2_]	*Z* = 2
*M**_r_* = 849.12	*F*(000) = 868
Triclinic, *P*1	*D*_x_ = 1.446 Mg m^−^^3^
Hall symbol: -P 1	Mo *K*α radiation, λ = 0.71073 Å
*a* = 10.5685 (10) Å	Cell parameters from 2285 reflections
*b* = 12.5224 (11) Å	θ = 2.2–20.7°
*c* = 16.3983 (14) Å	µ = 0.58 mm^−^^1^
α = 82.088 (2)°	*T* = 150 K
β = 77.476 (2)°	Block, orange
γ = 67.212 (2)°	0.16 × 0.15 × 0.10 mm
*V* = 1949.7 (3) Å^3^	

### Data collection

**Table:**

Bruker SMART APEX CCD area-detector diffractometer	8942 independent reflections
Radiation source: fine-focus sealed tube	6714 reflections with *I* > 2σ(*I*)
graphite	*R*_int_ = 0.075
ω scans	θ_max_ = 27.5°, θ_min_ = 1.3°
Absorption correction: multi-scan (*SADABS*; Bruker, 2007)	*h* = −13→13
*T*_min_ = 0.913, *T*_max_ = 0.944	*k* = −16→16
25423 measured reflections	*l* = −21→21

### Refinement

**Table:**

Refinement on *F*^2^	Primary atom site location: structure-invariant direct methods
Least-squares matrix: full	Secondary atom site location: difference Fourier map
*R*[*F*^2^ > 2σ(*F*^2^)] = 0.053	Hydrogen site location: inferred from neighbouring sites
*wR*(*F*^2^) = 0.128	H-atom parameters constrained
*S* = 1.00	*w* = 1/[σ^2^(*F*_o_^2^) + (0.0566*P*)^2^] where *P* = (*F*_o_^2^ + 2*F*_c_^2^)/3
8942 reflections	(Δ/σ)_max_ = 0.001
478 parameters	Δρ_max_ = 1.02 e Å^−^^3^
3 restraints	Δρ_min_ = −0.81 e Å^−^^3^

### Special details

**Table:**

Geometry. All e.s.d.'s (except the e.s.d. in the dihedral angle between two l.s. planes) are estimated using the full covariance matrix. The cell e.s.d.'s are taken into account individually in the estimation of e.s.d.'s in distances, angles and torsion angles; correlations between e.s.d.'s in cell parameters are only used when they are defined by crystal symmetry. An approximate (isotropic) treatment of cell e.s.d.'s is used for estimating e.s.d.'s involving l.s. planes.
Refinement. Refinement of *F*^2^ against ALL reflections. The weighted *R*-factor *wR* and goodness of fit *S* are based on *F*^2^, conventional *R*-factors *R* are based on *F*, with *F* set to zero for negative *F*^2^. The threshold expression of *F*^2^ > σ(*F*^2^) is used only for calculating *R*-factors(gt) *etc*. and is not relevant to the choice of reflections for refinement. *R*-factors based on *F*^2^ are statistically about twice as large as those based on *F*, and *R*- factors based on ALL data will be even larger.

### Fractional atomic coordinates and isotropic or equivalent isotropic displacement parameters (Å^2^)

**Table:**

	*x*	*y*	*z*	*U*_iso_*/*U*_eq_	
Mo1	0.77982 (3)	0.84316 (3)	0.741327 (19)	0.01739 (10)	
Cl1	1.00258 (9)	0.78068 (8)	0.79778 (6)	0.0237 (2)	
P1	0.77983 (9)	1.04763 (8)	0.71651 (6)	0.0187 (2)	
P2	0.80792 (10)	0.63059 (8)	0.77147 (6)	0.0218 (2)	
S1	0.61369 (10)	0.87871 (8)	0.88984 (6)	0.0247 (2)	
C1	0.6626 (4)	0.8368 (3)	0.6676 (2)	0.0246 (8)	
C2	0.8831 (4)	0.8414 (3)	0.6237 (2)	0.0259 (8)	
C3	0.5806 (4)	0.9314 (3)	0.7956 (2)	0.0212 (8)	
C4	0.3344 (4)	1.0250 (3)	0.8332 (2)	0.0261 (9)	
C5	0.2484 (4)	0.9681 (3)	0.8282 (2)	0.0284 (9)	
H5	0.2768	0.9084	0.7905	0.034*	
C6	0.1185 (4)	1.0009 (4)	0.8801 (3)	0.0338 (10)	
H6	0.0563	0.9636	0.8782	0.041*	
C7	0.0797 (4)	1.0870 (4)	0.9344 (3)	0.0381 (10)	
H7	−0.0100	1.1102	0.9688	0.046*	
C8	0.1699 (5)	1.1401 (4)	0.9392 (3)	0.0389 (11)	
H8	0.1431	1.1980	0.9780	0.047*	
C9	0.3002 (4)	1.1094 (4)	0.8875 (3)	0.0337 (10)	
H9	0.3631	1.1458	0.8899	0.040*	
C10	0.9438 (4)	1.0723 (3)	0.6985 (2)	0.0213 (8)	
C11	1.0648 (4)	0.9969 (3)	0.6529 (2)	0.0260 (8)	
H11	1.0672	0.9253	0.6382	0.031*	
C12	1.1829 (4)	1.0261 (4)	0.6287 (3)	0.0362 (10)	
H12	1.2651	0.9746	0.5970	0.043*	
C13	1.1808 (4)	1.1293 (4)	0.6504 (3)	0.0367 (10)	
H13	1.2611	1.1491	0.6332	0.044*	
C14	1.0628 (4)	1.2033 (4)	0.6969 (3)	0.0390 (11)	
H14	1.0618	1.2738	0.7129	0.047*	
C15	0.9447 (4)	1.1748 (3)	0.7206 (3)	0.0317 (9)	
H15	0.8631	1.2265	0.7526	0.038*	
C16	0.6791 (4)	1.1418 (3)	0.8016 (2)	0.0190 (7)	
C17	0.5762 (4)	1.2503 (3)	0.7907 (2)	0.0257 (8)	
H17	0.5522	1.2773	0.7370	0.031*	
C18	0.5087 (4)	1.3192 (3)	0.8585 (3)	0.0319 (9)	
H18	0.4391	1.3936	0.8506	0.038*	
C19	0.5411 (4)	1.2812 (3)	0.9366 (2)	0.0296 (9)	
H19	0.4936	1.3285	0.9827	0.036*	
C20	0.6439 (4)	1.1730 (3)	0.9479 (2)	0.0269 (9)	
H20	0.6674	1.1465	1.0017	0.032*	
C21	0.7121 (4)	1.1037 (3)	0.8810 (2)	0.0233 (8)	
H21	0.7820	1.0296	0.8893	0.028*	
C22	0.7063 (4)	1.1261 (3)	0.6235 (2)	0.0208 (8)	
C23	0.5875 (4)	1.1172 (3)	0.6072 (2)	0.0258 (8)	
H23	0.5432	1.0723	0.6451	0.031*	
C24	0.5325 (4)	1.1720 (3)	0.5373 (2)	0.0292 (9)	
H24	0.4511	1.1644	0.5274	0.035*	
C25	0.5952 (4)	1.2384 (3)	0.4810 (2)	0.0293 (9)	
H25	0.5569	1.2767	0.4328	0.035*	
C26	0.7136 (4)	1.2480 (4)	0.4960 (3)	0.0349 (10)	
H26	0.7577	1.2925	0.4577	0.042*	
C27	0.7684 (4)	1.1931 (3)	0.5667 (2)	0.0298 (9)	
H27	0.8495	1.2011	0.5766	0.036*	
C28	0.6717 (4)	0.5957 (3)	0.7413 (2)	0.0262 (9)	
C29	0.6950 (5)	0.5153 (3)	0.6840 (3)	0.0330 (10)	
H29	0.7876	0.4705	0.6591	0.040*	
C30	0.5822 (6)	0.5006 (4)	0.6631 (3)	0.0454 (13)	
H30	0.5983	0.4467	0.6229	0.054*	
C31	0.4482 (6)	0.5630 (4)	0.6998 (3)	0.0488 (14)	
H31	0.3720	0.5528	0.6846	0.059*	
C32	0.4239 (5)	0.6405 (4)	0.7588 (3)	0.0418 (12)	
H32	0.3313	0.6822	0.7853	0.050*	
C33	0.5353 (4)	0.6575 (4)	0.7793 (3)	0.0333 (10)	
H33	0.5185	0.7115	0.8195	0.040*	
C34	0.8098 (4)	0.5616 (3)	0.8780 (2)	0.0250 (8)	
C35	0.7853 (5)	0.4590 (4)	0.8978 (3)	0.0403 (11)	
H35	0.7664	0.4240	0.8563	0.048*	
C36	0.7882 (5)	0.4078 (4)	0.9780 (3)	0.0437 (12)	
H36	0.7733	0.3368	0.9908	0.052*	
C37	0.8122 (4)	0.4579 (4)	1.0391 (3)	0.0352 (10)	
H37	0.8138	0.4223	1.0941	0.042*	
C38	0.8340 (4)	0.5609 (4)	1.0196 (2)	0.0317 (9)	
H38	0.8488	0.5972	1.0619	0.038*	
C39	0.8346 (4)	0.6120 (3)	0.9393 (2)	0.0267 (8)	
H39	0.8522	0.6819	0.9264	0.032*	
C40	0.9734 (4)	0.5414 (3)	0.7104 (3)	0.0301 (9)	
C41	1.0837 (4)	0.4694 (4)	0.7479 (3)	0.0394 (11)	
H41	1.0720	0.4585	0.8072	0.047*	
C42	1.2122 (5)	0.4129 (4)	0.6989 (4)	0.0572 (16)	
H42	1.2880	0.3632	0.7251	0.069*	
C43	1.2308 (6)	0.4275 (5)	0.6144 (4)	0.0636 (18)	
H43	1.3191	0.3879	0.5816	0.076*	
C44	1.1228 (6)	0.4992 (5)	0.5763 (4)	0.0601 (16)	
H44	1.1361	0.5092	0.5169	0.072*	
C45	0.9937 (5)	0.5577 (4)	0.6235 (3)	0.0417 (11)	
H45	0.9194	0.6087	0.5967	0.050*	
O1	0.5958 (3)	0.8323 (3)	0.62133 (18)	0.0408 (8)	
O2	0.9334 (3)	0.8416 (3)	0.55439 (18)	0.0425 (8)	
O3	0.4605 (3)	0.9978 (2)	0.77308 (16)	0.0340 (7)	

### Atomic displacement parameters (Å^2^)

**Table:**

	*U*^11^	*U*^22^	*U*^33^	*U*^12^	*U*^13^	*U*^23^
Mo1	0.01565 (16)	0.02145 (17)	0.01619 (16)	−0.00996 (12)	−0.00020 (11)	0.00036 (12)
Cl1	0.0189 (4)	0.0264 (5)	0.0288 (5)	−0.0124 (4)	−0.0075 (4)	0.0054 (4)
P1	0.0146 (4)	0.0212 (5)	0.0189 (5)	−0.0077 (4)	0.0006 (4)	0.0003 (4)
P2	0.0221 (5)	0.0220 (5)	0.0231 (5)	−0.0111 (4)	−0.0021 (4)	−0.0015 (4)
S1	0.0223 (5)	0.0310 (5)	0.0189 (4)	−0.0107 (4)	−0.0001 (4)	0.0018 (4)
C1	0.025 (2)	0.034 (2)	0.0206 (19)	−0.0184 (17)	−0.0052 (14)	0.0052 (16)
C2	0.026 (2)	0.033 (2)	0.0200 (13)	−0.0162 (17)	0.0028 (13)	−0.0023 (16)
C3	0.0212 (19)	0.0235 (19)	0.0226 (19)	−0.0149 (16)	−0.0007 (15)	0.0014 (15)
C4	0.0122 (17)	0.039 (2)	0.022 (2)	−0.0074 (16)	0.0004 (15)	0.0017 (17)
C5	0.023 (2)	0.033 (2)	0.028 (2)	−0.0094 (17)	−0.0037 (17)	−0.0026 (17)
C6	0.023 (2)	0.047 (3)	0.034 (2)	−0.020 (2)	−0.0021 (18)	0.002 (2)
C7	0.023 (2)	0.048 (3)	0.037 (3)	−0.014 (2)	0.0089 (19)	−0.005 (2)
C8	0.040 (3)	0.037 (2)	0.040 (3)	−0.017 (2)	0.003 (2)	−0.012 (2)
C9	0.029 (2)	0.042 (3)	0.035 (2)	−0.022 (2)	0.0000 (18)	−0.0013 (19)
C10	0.0200 (19)	0.0242 (19)	0.0209 (19)	−0.0115 (16)	−0.0033 (15)	0.0044 (15)
C11	0.0203 (19)	0.033 (2)	0.027 (2)	−0.0147 (17)	0.0024 (16)	−0.0052 (17)
C12	0.021 (2)	0.043 (3)	0.039 (3)	−0.0114 (19)	0.0069 (18)	−0.006 (2)
C13	0.027 (2)	0.044 (3)	0.043 (3)	−0.024 (2)	0.0030 (19)	0.005 (2)
C14	0.034 (2)	0.031 (2)	0.056 (3)	−0.021 (2)	0.002 (2)	−0.006 (2)
C15	0.021 (2)	0.030 (2)	0.043 (3)	−0.0130 (17)	0.0035 (18)	−0.0036 (18)
C16	0.0162 (17)	0.0236 (19)	0.0172 (18)	−0.0119 (15)	0.0047 (14)	−0.0001 (14)
C17	0.022 (2)	0.025 (2)	0.027 (2)	−0.0099 (16)	0.0016 (16)	0.0030 (16)
C18	0.027 (2)	0.026 (2)	0.035 (2)	−0.0070 (17)	0.0031 (18)	−0.0023 (18)
C19	0.029 (2)	0.031 (2)	0.027 (2)	−0.0135 (18)	0.0081 (17)	−0.0092 (17)
C20	0.025 (2)	0.036 (2)	0.0205 (19)	−0.0168 (18)	0.0035 (16)	−0.0015 (17)
C21	0.0209 (19)	0.0223 (19)	0.028 (2)	−0.0113 (16)	−0.0017 (16)	0.0010 (16)
C22	0.0177 (18)	0.0218 (19)	0.0194 (18)	−0.0062 (15)	0.0018 (14)	−0.0017 (15)
C23	0.0180 (19)	0.032 (2)	0.026 (2)	−0.0099 (16)	−0.0023 (16)	0.0037 (17)
C24	0.023 (2)	0.034 (2)	0.028 (2)	−0.0105 (18)	−0.0026 (17)	0.0015 (17)
C25	0.036 (2)	0.029 (2)	0.0159 (19)	−0.0071 (18)	−0.0046 (17)	0.0042 (16)
C26	0.039 (3)	0.036 (2)	0.030 (2)	−0.021 (2)	−0.0022 (19)	0.0094 (19)
C27	0.027 (2)	0.035 (2)	0.027 (2)	−0.0164 (18)	0.0008 (17)	0.0050 (17)
C28	0.033 (2)	0.028 (2)	0.024 (2)	−0.0200 (18)	−0.0096 (17)	0.0094 (16)
C29	0.046 (3)	0.031 (2)	0.033 (2)	−0.025 (2)	−0.013 (2)	0.0047 (18)
C30	0.074 (4)	0.042 (3)	0.041 (3)	−0.040 (3)	−0.029 (3)	0.015 (2)
C31	0.068 (4)	0.049 (3)	0.056 (3)	−0.048 (3)	−0.039 (3)	0.030 (3)
C32	0.036 (3)	0.044 (3)	0.054 (3)	−0.027 (2)	−0.016 (2)	0.018 (2)
C33	0.033 (2)	0.036 (2)	0.038 (2)	−0.022 (2)	−0.0061 (19)	0.0023 (19)
C34	0.023 (2)	0.025 (2)	0.025 (2)	−0.0087 (16)	−0.0032 (16)	−0.0002 (16)
C35	0.054 (3)	0.035 (2)	0.041 (3)	−0.024 (2)	−0.017 (2)	0.008 (2)
C36	0.052 (3)	0.035 (3)	0.048 (3)	−0.027 (2)	−0.013 (2)	0.019 (2)
C37	0.031 (2)	0.040 (3)	0.030 (2)	−0.013 (2)	−0.0043 (19)	0.0127 (19)
C38	0.028 (2)	0.040 (2)	0.024 (2)	−0.0104 (19)	−0.0035 (17)	−0.0001 (18)
C39	0.022 (2)	0.026 (2)	0.028 (2)	−0.0077 (16)	−0.0016 (16)	0.0017 (16)
C40	0.030 (2)	0.029 (2)	0.035 (2)	−0.0169 (18)	0.0023 (18)	−0.0126 (18)
C41	0.024 (2)	0.037 (2)	0.062 (3)	−0.0143 (19)	−0.005 (2)	−0.015 (2)
C42	0.026 (2)	0.042 (3)	0.112 (5)	−0.017 (2)	−0.002 (3)	−0.030 (3)
C43	0.037 (3)	0.052 (3)	0.102 (5)	−0.027 (3)	0.032 (3)	−0.046 (3)
C44	0.066 (4)	0.056 (3)	0.061 (4)	−0.039 (3)	0.036 (3)	−0.037 (3)
C45	0.048 (3)	0.039 (3)	0.038 (3)	−0.023 (2)	0.011 (2)	−0.012 (2)
O1	0.052 (2)	0.056 (2)	0.0298 (17)	−0.0332 (17)	−0.0188 (15)	0.0068 (14)
O2	0.0468 (19)	0.059 (2)	0.0250 (16)	−0.0300 (16)	0.0082 (14)	−0.0066 (14)
O3	0.0160 (14)	0.0525 (18)	0.0246 (15)	−0.0089 (13)	−0.0003 (11)	0.0103 (13)

### Geometric parameters (Å, °)

**Table:**

Mo1—C1	1.938 (4)	C20—C21	1.381 (5)
Mo1—C2	1.998 (4)	C20—H20	0.9500
Mo1—C3	2.025 (4)	C21—H21	0.9500
Mo1—Cl1	2.5160 (9)	C22—C23	1.387 (5)
Mo1—P1	2.5368 (10)	C22—C27	1.397 (5)
Mo1—P2	2.5509 (10)	C23—C24	1.373 (5)
Mo1—S1	2.6553 (10)	C23—H23	0.9500
P1—C16	1.819 (4)	C24—C25	1.391 (5)
P1—C10	1.831 (4)	C24—H24	0.9500
P1—C22	1.845 (4)	C25—C26	1.378 (6)
P2—C40	1.829 (4)	C25—H25	0.9500
P2—C28	1.834 (4)	C26—C27	1.383 (5)
P2—C34	1.840 (4)	C26—H26	0.9500
S1—C3	1.650 (4)	C27—H27	0.9500
C1—O1	1.163 (4)	C28—C29	1.388 (5)
C2—O2	1.146 (4)	C28—C33	1.394 (6)
C3—O3	1.319 (4)	C29—C30	1.392 (6)
C4—C9	1.365 (6)	C29—H29	0.9500
C4—C5	1.375 (5)	C30—C31	1.372 (7)
C4—O3	1.427 (4)	C30—H30	0.9500
C5—C6	1.390 (5)	C31—C32	1.379 (7)
C5—H5	0.9500	C31—H31	0.9500
C6—C7	1.374 (6)	C32—C33	1.389 (5)
C6—H6	0.9500	C32—H32	0.9500
C7—C8	1.376 (6)	C33—H33	0.9500
C7—H7	0.9500	C34—C39	1.377 (5)
C8—C9	1.392 (6)	C34—C35	1.390 (5)
C8—H8	0.9500	C35—C36	1.382 (6)
C9—H9	0.9500	C35—H35	0.9500
C10—C15	1.385 (5)	C36—C37	1.367 (6)
C10—C11	1.389 (5)	C36—H36	0.9500
C11—C12	1.395 (5)	C37—C38	1.380 (6)
C11—H11	0.9500	C37—H37	0.9500
C12—C13	1.378 (6)	C38—C39	1.383 (5)
C12—H12	0.9500	C38—H38	0.9500
C13—C14	1.372 (6)	C39—H39	0.9500
C13—H13	0.9500	C40—C41	1.377 (6)
C14—C15	1.391 (5)	C40—C45	1.392 (6)
C14—H14	0.9500	C41—C42	1.389 (6)
C15—H15	0.9500	C41—H41	0.9500
C16—C17	1.391 (5)	C42—C43	1.354 (8)
C16—C21	1.393 (5)	C42—H42	0.9500
C17—C18	1.391 (5)	C43—C44	1.364 (8)
C17—H17	0.9500	C43—H43	0.9500
C18—C19	1.372 (5)	C44—C45	1.386 (6)
C18—H18	0.9500	C44—H44	0.9500
C19—C20	1.388 (5)	C45—H45	0.9500
C19—H19	0.9500		
			
C1—Mo1—C2	71.73 (15)	C17—C18—H18	119.6
C1—Mo1—C3	73.73 (15)	C18—C19—C20	119.5 (4)
C2—Mo1—C3	132.98 (15)	C18—C19—H19	120.3
C1—Mo1—Cl1	154.93 (12)	C20—C19—H19	120.3
C2—Mo1—Cl1	91.25 (11)	C21—C20—C19	120.3 (4)
C3—Mo1—Cl1	130.01 (10)	C21—C20—H20	119.9
C1—Mo1—P1	104.78 (11)	C19—C20—H20	119.9
C2—Mo1—P1	78.19 (11)	C20—C21—C16	120.4 (3)
C3—Mo1—P1	80.87 (10)	C20—C21—H21	119.8
Cl1—Mo1—P1	88.95 (3)	C16—C21—H21	119.8
C1—Mo1—P2	81.36 (11)	C23—C22—C27	117.9 (3)
C2—Mo1—P2	101.33 (11)	C23—C22—P1	119.9 (3)
C3—Mo1—P2	103.97 (10)	C27—C22—P1	122.2 (3)
Cl1—Mo1—P2	84.26 (3)	C24—C23—C22	121.3 (4)
P1—Mo1—P2	173.19 (3)	C24—C23—H23	119.3
C1—Mo1—S1	104.16 (11)	C22—C23—H23	119.3
C2—Mo1—S1	170.67 (11)	C23—C24—C25	120.4 (4)
C3—Mo1—S1	38.38 (10)	C23—C24—H24	119.8
Cl1—Mo1—S1	95.19 (3)	C25—C24—H24	119.8
P1—Mo1—S1	95.15 (3)	C26—C25—C24	119.1 (4)
P2—Mo1—S1	86.06 (3)	C26—C25—H25	120.4
C16—P1—C10	100.80 (16)	C24—C25—H25	120.4
C16—P1—C22	104.60 (16)	C25—C26—C27	120.4 (4)
C10—P1—C22	101.17 (16)	C25—C26—H26	119.8
C16—P1—Mo1	114.53 (11)	C27—C26—H26	119.8
C10—P1—Mo1	120.45 (12)	C26—C27—C22	120.9 (4)
C22—P1—Mo1	113.17 (12)	C26—C27—H27	119.5
C40—P2—C28	106.39 (18)	C22—C27—H27	119.5
C40—P2—C34	104.36 (18)	C29—C28—C33	119.2 (4)
C28—P2—C34	100.80 (17)	C29—C28—P2	125.1 (3)
C40—P2—Mo1	108.48 (13)	C33—C28—P2	115.7 (3)
C28—P2—Mo1	113.81 (12)	C28—C29—C30	119.7 (4)
C34—P2—Mo1	121.70 (12)	C28—C29—H29	120.1
C3—S1—Mo1	49.67 (13)	C30—C29—H29	120.1
O1—C1—Mo1	177.8 (3)	C31—C30—C29	120.7 (5)
O2—C2—Mo1	175.0 (3)	C31—C30—H30	119.6
O3—C3—S1	129.0 (3)	C29—C30—H30	119.6
O3—C3—Mo1	138.8 (3)	C30—C31—C32	120.1 (4)
S1—C3—Mo1	91.95 (16)	C30—C31—H31	120.0
C9—C4—C5	123.6 (4)	C32—C31—H31	120.0
C9—C4—O3	120.1 (3)	C31—C32—C33	119.8 (5)
C5—C4—O3	116.1 (3)	C31—C32—H32	120.1
C4—C5—C6	117.7 (4)	C33—C32—H32	120.1
C4—C5—H5	121.2	C32—C33—C28	120.4 (4)
C6—C5—H5	121.2	C32—C33—H33	119.8
C7—C6—C5	120.2 (4)	C28—C33—H33	119.8
C7—C6—H6	119.9	C39—C34—C35	119.2 (4)
C5—C6—H6	119.9	C39—C34—P2	120.0 (3)
C6—C7—C8	120.5 (4)	C35—C34—P2	120.7 (3)
C6—C7—H7	119.7	C36—C35—C34	120.0 (4)
C8—C7—H7	119.7	C36—C35—H35	120.0
C7—C8—C9	120.4 (4)	C34—C35—H35	120.0
C7—C8—H8	119.8	C37—C36—C35	120.9 (4)
C9—C8—H8	119.8	C37—C36—H36	119.5
C4—C9—C8	117.6 (4)	C35—C36—H36	119.5
C4—C9—H9	121.2	C36—C37—C38	119.0 (4)
C8—C9—H9	121.2	C36—C37—H37	120.5
C15—C10—C11	118.5 (3)	C38—C37—H37	120.5
C15—C10—P1	120.0 (3)	C37—C38—C39	120.9 (4)
C11—C10—P1	121.0 (3)	C37—C38—H38	119.6
C10—C11—C12	120.2 (4)	C39—C38—H38	119.6
C10—C11—H11	119.9	C34—C39—C38	120.0 (4)
C12—C11—H11	119.9	C34—C39—H39	120.0
C13—C12—C11	120.3 (4)	C38—C39—H39	120.0
C13—C12—H12	119.9	C41—C40—C45	119.0 (4)
C11—C12—H12	119.9	C41—C40—P2	121.7 (3)
C14—C13—C12	120.1 (4)	C45—C40—P2	118.7 (3)
C14—C13—H13	120.0	C40—C41—C42	119.8 (5)
C12—C13—H13	120.0	C40—C41—H41	120.1
C13—C14—C15	119.7 (4)	C42—C41—H41	120.1
C13—C14—H14	120.1	C43—C42—C41	120.9 (5)
C15—C14—H14	120.1	C43—C42—H42	119.5
C10—C15—C14	121.2 (4)	C41—C42—H42	119.5
C10—C15—H15	119.4	C42—C43—C44	120.0 (5)
C14—C15—H15	119.4	C42—C43—H43	120.0
C17—C16—C21	119.1 (3)	C44—C43—H43	120.0
C17—C16—P1	123.5 (3)	C43—C44—C45	120.4 (5)
C21—C16—P1	117.4 (3)	C43—C44—H44	119.8
C18—C17—C16	119.8 (4)	C45—C44—H44	119.8
C18—C17—H17	120.1	C44—C45—C40	119.8 (5)
C16—C17—H17	120.1	C44—C45—H45	120.1
C19—C18—C17	120.9 (4)	C40—C45—H45	120.1
C19—C18—H18	119.6	C3—O3—C4	120.2 (3)

### Hydrogen-bond geometry (Å, °)

**Table:**

Cg1, Cg2, Cg3 and Cg7 are the centroids of the C4–C9, C10–C15, C16–C21 and C40–C45 rings, respectively.

**Table:**

*D*—H···*A*	*D*—H	H···*A*	*D*···*A*	*D*—H···*A*
C23—H23···O3	0.95	2.31	3.208 (5)	157
C24—H24···O1^i^	0.95	2.58	3.199 (5)	123
C39—H39···Cl1	0.95	2.80	3.573 (4)	139
C39—H39···S1	0.95	2.87	3.361 (4)	114
C9—H9···Cg3	0.95	2.97	3.896 (5)	165
C14—H14···Cg7^ii^	0.95	2.83	3.663 (5)	147
C20—H20···Cg1^iii^	0.95	2.97	3.802 (4)	147
C27—H27···Cg2	0.95	2.84	3.636 (5)	141

Symmetry codes: (i) −*x*+1, −*y*+2, −*z*+1; (ii) *x*, *y*+1, *z*; (iii) −*x*+1, −*y*+2, −*z*+2.
